# Functionalizing Injectable Hydrogels with Cobalt‐Based Metallacarboranes for Targeted Delivery in Triple‐Negative Breast Cancer

**DOI:** 10.1002/cbic.202500589

**Published:** 2025-10-07

**Authors:** Neville Murphy, Roberto González‐Gómez, Nivethitha Ashok, Enda O’Connell, Howard Fearnhead, William J. Tipping, Karen Faulds, Wenming Tong, Abhay Pandit, Róisín M. Dwyer, Duncan Graham, Pau Farràs

**Affiliations:** ^1^ School of Biological and Chemical Sciences Ryan Institute University of Galway H91 CF50 Galway Ireland; ^2^ CÚRAM Research Ireland Centre for Medical Devices University of Galway H91 W2TY Galway Ireland; ^3^ Genomics and Screening Core University of Galway H91 W2TY Galway Ireland; ^4^ Pharmacology and Therapeutics University of Galway H91 TK33 Galway Ireland; ^5^ Centre for Molecular Nanometrology Department of Pure and Applied Chemistry Technology and Innovation Centre University of Strathclyde Glasgow G1 1RD UK; ^6^ Lambe Institute for Translational Research School of Medicine University of Galway H91 TK33 Galway Ireland

**Keywords:** cobalt–bis(dicarbollide), hyaluronic acid, metallacarboranes, targeted delivery systems, triple‐negative breast cancer

## Abstract

Cobalt‐based metallacarboranes have emerged as potential candidates for cancer treatment owing to their unique structural properties. In this study, a biocompatible delivery platform is developed by noncovalently incorporating cobalt metallacarborane (CoSAN) into hyaluronic acid (HA) functionalized with lysine (Lys). HA‐Lys **2** enables the electrostatic interaction of CoSAN while retaining its cytotoxic activity, as confirmed by cellular assays using MDA‐MB‐231 triple‐negative breast cancer cells. Elemental mapping via energy‐dispersive X‐ray spectroscopy (EDX) confirms the successful and homogeneous incorporation of CoSAN to lead HA‐Lys‐CoSAN **3**, and the composite is further characterized using diffusion‐ordered nuclear magnetic resonance (NMR) spectroscopy (DOSY). Stimulated Raman scattering (SRS) microscopy data demonstrate comparable cellular uptake in MDA‐MB‐231 cells of free and HA‐loaded CoSAN. Additionally, release studies under physiologically relevant conditions show a sustained release profile over 24 h with pH dependency to mimic normal and tumor microenvironments. The present study describes a viable method for integrating metallacarboranes into a polymeric drug delivery system without compromising their anticancer properties, thereby advancing their potential for future therapeutic use.

## Graphical abstract

This study reports a biocompatible HA–lysine hydrogel loaded with cobalt metallacarborane (CoSAN) for targeted release into triple‐negative breast cancer cells. The system maintains CoSAN cytotoxicity, as confirmed by cell assays and imaging, representing an attractive approach toward a localised TNBC treatment.

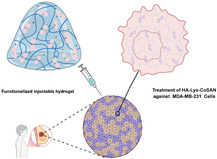

## 1 Introduction

Triple‐negative breast cancer (TNBC), which accounts for 15%–20% of all breast cancer cases, is uniquely characterized by the absence of estrogen, progesterone, and human epidermal growth factor receptor 2 (HER‐2) receptors, making it resistant to most targeted therapies.^[^ ^1^ ^]^ Despite the initial response to chemotherapy, TNBC has a poor prognosis, as treatment efficacy is often compromised by the rapid development of drug resistance and consequent metastasis,^[^ ^2^, ^3^, ^4^ ^–^ ^5^ ^]^ which has led to the design of novel inorganic compounds capable of serving as next‐generation cancer therapeutics.^[^ ^6^ ^]^ Over the past few decades, carboranes, polyhedral boranes in which a BH unit is replaced with an isoelectronic CH unit, have been investigated predominantly in the context of Boron Neutron Capture Therapy (BNCT), where the main objective is to localize the highest amount of boron atoms near tumor cells and, in general, aiming at using nontoxic boron clusters.^[^ ^7^ ^]^ More recently, boranes and carboranes have also emerged as lead compounds as potential drugs, antineoplastic/cytotoxic agents, estrogen agonists and antagonists,^[^ ^8^ ^]^ and immunomodulators,^[^ ^9^ ^]^ owing to their chemical versatility, ability to form stable covalent bonds with biological targets, and influence on key cellular pathways involved in cellular modulation. Most of these applications come from bioactive drugs covalently bound to the boron clusters. Inorganic boron clusters assemble into 3D polyhedral configurations via characteristic three‐center, two‐electron (3*c*–2*e*) *σ‐*bonding.^[^ ^10^ ^,^ ^11^ ^]^ Their cage‐like structure confers chemical and thermal stability, versatile functionalization, and typically low acute biological toxicity, making them promising candidates for biomedical applications.^[^ ^12^, ^13^ ^–^ ^14^ ^]^

Metallacarboranes are compounds that contain one or more metal atoms and one or more boron hydrides, and the reactive BH and CH (in case of carborane cages) vertices trigger unconventional interactions with neighboring molecules.^[^ ^14^, ^15^, ^16^, ^17^ ^–^ ^18^ ^]^ Within the metallacarborane family, metallabis(dicarbollide) sandwich complexes consisting of a metal center flanked by two [C_2_B_9_H_11_]^2−^ dicarbollide ligands have attracted attention for their remarkable biological activity against cancer cells. These species are commonly denoted by the metal symbol followed by “SAN”, reflecting their sandwich architecture; for example, cobalt–bis(dicarbollide) is recognized as **[CoSAN]** ^−^ ([3,3′–Co(1,2‐C_2_B_9_H_11_)_2_]^−^). CoSAN's structural rigidity and 3D *σ‐*aromaticity confer high resistance in physiological media and resist DNA interactions, indicating favorable persistence in biological media without rapid breakdown, a desired property for cancer therapies.^[^ ^19^, ^20^, ^21^ ^–^ ^22^ ^]^ In addition, reports on the integration of (metalla)carboranes with organic biomolecules have shown that hybrid constructs can be generated, combining and often enhancing the complementary features of both components, opening new avenues for the utilization of metallacarboranes in the medicinal field.^[^ ^15^ ^–^ ^18^ ^]^ Within CoSAN's structure, the BH vertex on the open phase coordinating to Co, opposite to the CH vertices (known as B(8) and B(8’) within the metallacarborane community), is the most reactive and allows for intermolecular interactions in aqueous solutions between different CoSAN units, forming aggregates in the form of micelles and vesicles.^[^ ^23^ ^]^ This self‐aggregation property, coming from its amphiphilic behavior, more recently named as chaotropic carriers, triggers its capacity to cross lipid bilayer membranes.^[^ ^24^ ^]^

A recent investigation by Bednarska–Szczepaniak et al. evaluated CoSAN, FeSAN, and CrSAN‐based metallacarboranes in A2780, A2780cis, and OVCAR‐3 ovarian cancer cell lines, with normal human dermal fibroblasts (HDFs) as controls. Colourimetric viability assays showed efficient cellular uptake of all three complexes, which markedly elevated mitochondrial activity and precipitated energy exhaustion‐mediated cell death. The iron analog produced the strongest cytotoxic response, while exhibiting lower toxicity toward HDFs, underscoring its therapeutic potential, highlighting the importance of investigating and understanding how the variations in the metal center influence anticancer efficacy.^[^ ^25^ ^]^ Our research group has reported a similar trend for TNBC MDA‐MB‐231 cell lines, showing an immediate drug uptake using stimulated Raman spectroscopy (SRS) imaging.^[^ ^12^ ^]^

Targeted drug delivery is a key challenge in cancer therapy, aimed at minimizing side effects and enhancing the therapeutic index of chemotherapeutic agents.^[^ ^26^ ^]^ Various approaches for incorporating carboranes into drug delivery systems, including liposomes,^[^ ^27^ ^]^ nanoparticles,^[^ ^28^ ^]^ and polycarbonate formulations,^[^ ^29^ ^]^ have been reported. Beyond polymeric and liposomal strategies, dendritic drug delivery systems have also been explored, where multiple *o*‐carborane clusters are combined with hydrophilic sugar moieties to create boron‐rich, tumor‐selective platforms. Carboranyl glycoconjugates bearing three, six, or nine glucose units exhibited pronounced cytotoxicity against MCF‐7 breast cancer cells, mediated through caspase‐3‐dependent apoptosis, underscoring the potential of carbohydrate‐functionalized carborane scaffolds for targeted therapy.^[^ ^30^ ^,^ ^31^ ^]^ However, research on delivery systems for metallacarboranes remains limited because their stability and biodistribution can be compromised by interactions with biological components, particularly serum proteins, which induce aggregation, nonspecific binding, and displacement from carriers.^[^ ^20^ ^,^ ^32^ ^]^ In particular, biodistribution studies reveal accumulation in reticuloendothelial system organs (liver, spleen, lungs) due to plasma protein aggregation, raising concerns for organ‐specific side effects.^[^ ^20^ ^,^ ^33^ ^]^ To address this gap, we investigated the loading of metallacarboranes onto hyaluronic acid (HA), a biocompatible carrier of choice, and to mitigate the adverse effects caused by the accumulation of CoSAN. HA has been extensively studied for drug delivery applications because of its versatility, biocompatibility, and biodegradability. Of particular interest in cancer therapy is HA's affinity for the CD44 receptor, which is overexpressed in many cancers of epithelial origin and is associated with various cancer‐related processes, including drug resistance through antiapoptotic signaling.^[^ ^34^ ^]^

**Figure 1:**
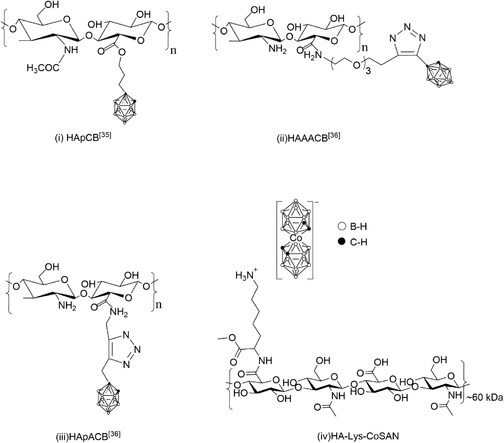
Schematic representations of i) HApCB (HA‐propylcarborane),^[^ ^35^ ^]^ ii) HApACB (hyaluronan‐propargylamido‐carborane),^[^ ^36^ ^]^ iii) HAAACB (hyaluronan‐amidoazido‐carborane),^[^ ^36^ ^]^ and iv) HA‐Lys‐CoSAN **3** (hyaluronan‐lysine‐metallacarborane, in this work).

Although examples of carborane‐loaded HA materials exist (**Figure** **1**), such as those reported by Di Meo et al.,^[^ ^35^ ^,^ ^36^ ^]^ their application as drug carriers is relatively rare. In their study, a series of novel hyaluronan (HA)‐carborane bioconjugates was synthesized and characterized as water‐soluble derivatives: HApCB(HA‐propylcarborane), HApACB (hyaluronan‐propargylamido‐carborane), and HAAACB (hyaluronan‐amidoazido‐carborane). These materials exhibited a strong affinity for the CD44 receptor in multiple cancer cell lines and enabled intracellular boron accumulation exceeding therapeutic thresholds, highlighting their potential as effective BNCT agents to accumulate higher doses of boron atoms.

Based on these insights, we developed a modified HA drug delivery system for metallacarboranes based on electrostatic interactions, leveraging HA's natural targeting capabilities to facilitate efficient delivery while preserving the structural integrity and biological activity of metallacarboranes. Lysine (Lys), a naturally occurring amino acid, was conjugated with the HA to produce the electrostatic interactions needed for the noncovalent loading of CoSAN. In addition, Lys plays a critical role in cell adhesion and collagen crosslinking and potentially influences the release properties of the system.^[^ ^37^ ^]^ We present a novel approach to enable selective delivery of CoSAN to cancer cells while preserving the compound's intrinsic bioactivity. The resulting HA‐Lys‐CoSAN **3** composite was systematically evaluated for structural integrity, cell viability, selectivity against HDF, cellular uptake, and release kinetics, confirming that CoSAN retains its biological activity upon incorporation into the HA‐Lys matrix.

## 2 Results and Discussion

### 2.1 Functionalisation of HA Biopolymers

**Figure 2:**
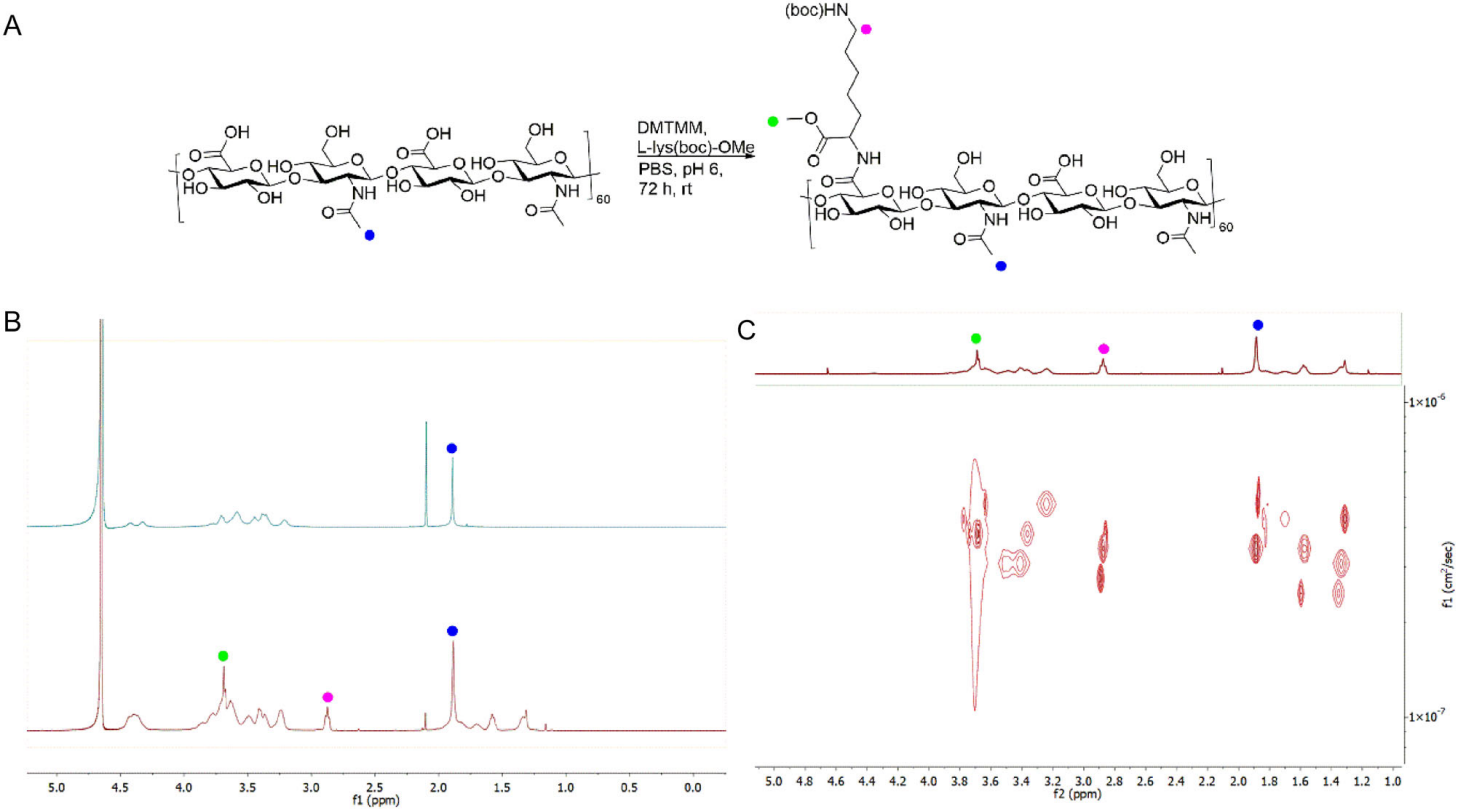
A) Reaction scheme for the addition of L‐lys(Boc)‐OMe to HA via amide coupling. B) ^1^H NMR spectra of HA (top) versus L‐Lys‐OMe modified HA **2** (bottom). C) DOSY spectrum of L‐Lys‐OMe modified HA **2**.

The first step of this study was to load CoSAN onto a HA‐based material using noncovalent bonding to facilitate the preparation of HA‐Lys‐CoSAN composites. To accomplish this, HA should be functionalized with a pendant group bearing a positive charge so that CoSAN (an anion) interacts via electrostatic forces with the HA‐functionalized backbone. HA was functionalized with lysine in the presence of phosphate‐buffered saline (PBS) (pH 6) at 30 °C overnight and then precipitated with ethanol. For this purpose, commercially available L‐lys(Boc)‐OMe, with the tert‐butyloxycarbonyl (Boc) protecting group that facilitates selective amide coupling on the amino acid backbone, and the methoxy group preventing polymerization, was selected, given the lack of toxicity and biological relevance as an amino acid derivative. Amide coupling was initially attempted with 1‐ethyl‐3‐(3‐dimethylaminopropyl)carbodiimide (EDC) and N‐hydroxysuccinimide (NHS) as coupling reagents at various pH values (5, 6, and 7.4) and equivalents of coupling reagents to L‐lysine/HA (0.6, 1, and 1.2), yielding no successful conjugation. The lack of reactivity led to the selection of 4‐(4,6‐Dimethoxy‐1,3,5‐triazin‐2‐yl)‐4‐methylmorpholinium chloride (DMTMM), which led to successful functionalization at pH 6, as shown in **Figure** **2**A.

Following functionalisation, the material was washed with MeOH to remove the coupling reagent and associated urea products, as well as unreacted L‐lys(Boc)‐OMe. The material was redissolved in a 50:50 1 M HCl:TFA solution and stirred at room temperature for 5 h to remove the Boc‐protecting group from the amine. A similar washing procedure, as described, was performed following neutralization. Characterization using ^1^H‐nuclear magnetic resonance (NMR) spectroscopy showed clear indications of successful modification, with the appearance of a singlet corresponding to the methoxy protecting groups, as well as the triplet of the 6‐CH_2_ (Figure 2B and Figure S2 and S3, Supporting Information), confirming incorporation into the polymer backbone. Fourier transform infrared spectroscopy (FTIR) also confirmed the hydrophilic nature of the HA‐Lys material with a broad peak centered at 3347 cm^*−*1^ corresponding to water molecules (O=H) in the structure of the polymer as well as the symmetric stretching vibrations of C=O present in the carboxylic acid groups. The amido group C=N is further confirmed by the presence of peaks at 1430 cm^*−*1^ (see Figure S3, Supporting Information). Diffusion‐ordered spectroscopy (DOSY) relates each resonance signal to its self‐diffusion coefficient using a pulsed gradient of a magnetic field. This helps to separate the components based on viscosity. It has been extensively used in the literature as a tool for calculating hydrodynamic radii, and monitoring the functionalisation of polymers.^[^ ^38^ ^]^ To further validate the lysine attenuation within the material, DOSY was performed. In our study, the lysine CH_2_ triplet at 2.88 ppm (3.46 × 10^7^ m^2^ s^*−*1^) exhibited diffusion values comparable to the HA CH_3_ singlet at 1.89 ppm (3.46 × 10^7^ m^2^ s^*−*1^), as shown in Figure 2C and S4, Supporting Information.

**Figure 3:**
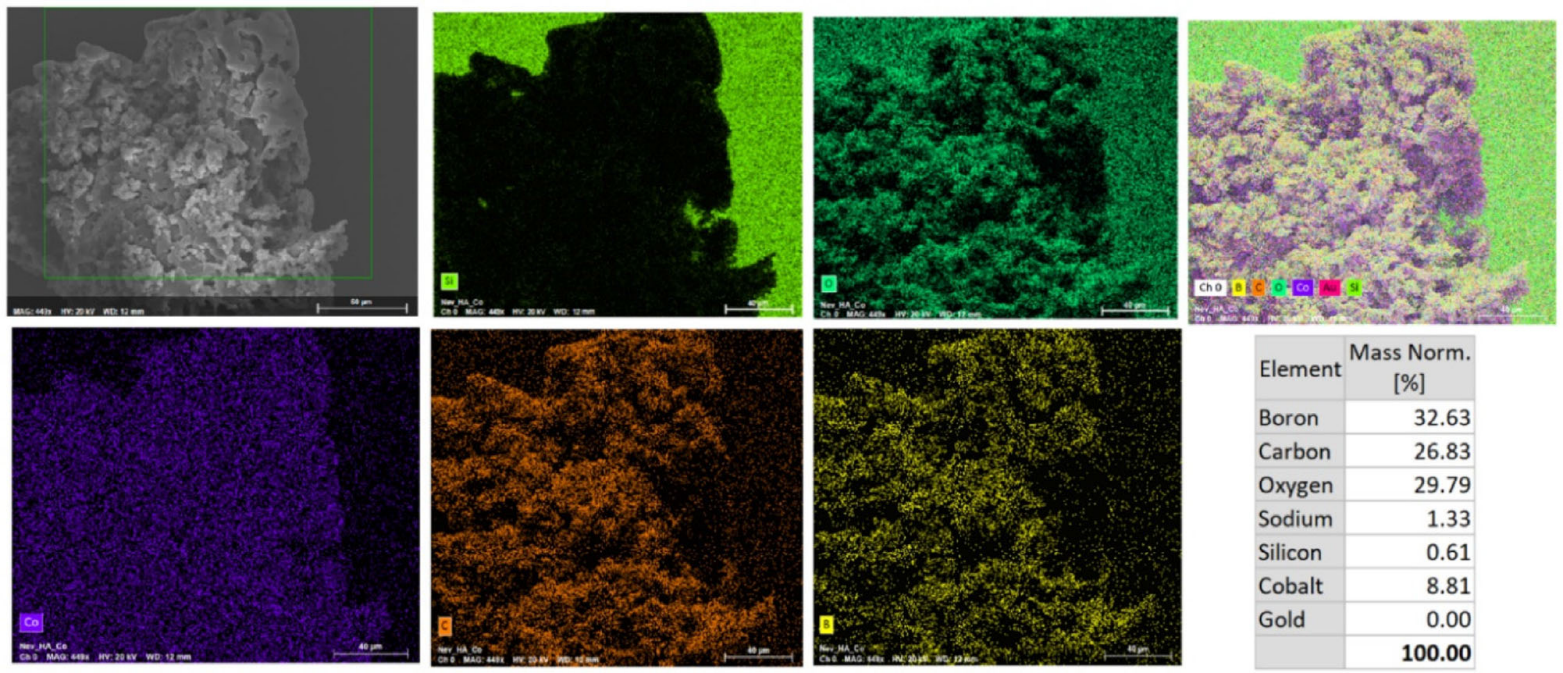
SEM images and EDX mapping of HA‐Lys‐CoSAN **3**.

To enhance the biomimicry of the material, the Boc protecting group was removed via trifluoroacetic acid (TFA) cleavage to yield a primary amine. This functional group serves as an anchoring site for metallacarborane loading through electrostatic interactions. The loaded material was then generated by solvation of the HA–lysine in 1 M HCl, followed by addition of a 1 mM aqueous solution of Na[CoSAN] to form a salt with the pendant lysine groups (synthesis and characterization are provided in Figure 1 and Figure S1, Supporting Information). This resulted in the precipitation of the polymer, which was washed with 1 M HCl to remove the unloaded CoSAN and then lyophilized. ^1^H‐NMR characterization of a D_2_O solution of **3** showed the presence of the expected C_c_=H from CoSAN at 4.42 ppm, a wide and undefined band between 3.1 and 1.2 ppm where B=H signals would appear, and the expected CH_2_ and CH_3_ units from HA (Figure S5, Supporting Information). ^11^B‐NMR also confirmed the presence of CoSAN with the expected symmetric peaks corresponding to the B=H vertices at 4.16, 0.56, −7.40, −18.11, and −23.85 ppm (Figure S6, Supporting Information). FTIR indicated the presence of B=H stretching with a clear peak centered at 2500 cm^−1^, along with the presence of other functional groups assigned to HA and Lys (Figure S8, Supporting Information). Further confirmation of CoSAN integration within HA‐Lys‐CoSAN **3** was carried out with EDX mapping (**Figure** **3**), which indicated sample homogeneity at the microscopic level. Boron and cobalt are both observed to be homogeneously distributed in regions with high carbon and oxygen content, consistent with the expected Co:B atomic ratio of 1:3.3 for COSAN (Co: 58.93 a.u.; B: 10.81 a.u.). The stated observations are further supported by scanning electron microscope (SEM) imaging, which visually confirms the porous nature of HA, even after the functionalisation with the CoSAN boron cages (Figure 3).

### 2.2 Cytotoxicity and Uptake Studies of Loaded HA

**Figure 4:**
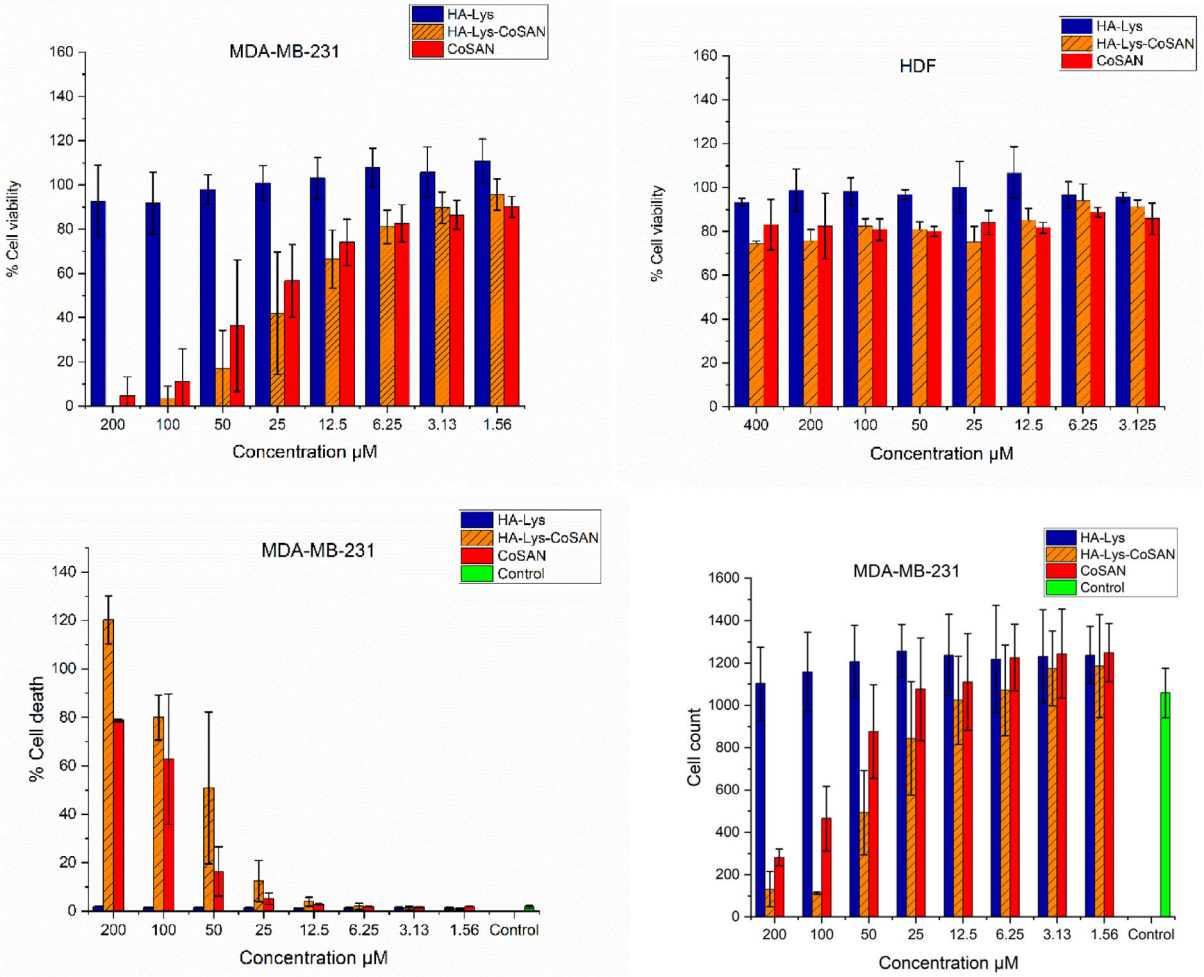
(top) Cell viability data of MDA‐MB‐231 and HDF cells following 72 h of incubation with the stated compounds at the stated concentrations. (bottom) Cell count and percentage of cell death of HA‐Lys‐CoSAN and CoSAN.

Building upon the successful chemical characterization of the HA‐Lys‐CoSAN material, its biological performance was evaluated using in vitro cell viability assays. This study aimed to investigate the cytotoxic effects of noncovalently loaded CoSAN into HA compared to those of CoSAN itself. The study was performed on the TNBC cell line MDA‐MB‐231 and normal HDF cell lines. This included incubation of MDA‐MB‐231 cells in dulbecco's modified eagle medium at 5% CO_2_, while HDF cells were maintained in medium lacking nonessential amino acids to simulate more physiologically relevant conditions. Additionally, all studies were performed in 384‐well plates seeded using the automated Multiflow^TM^ procedure to ensure reproducibility. The amount of loaded metallacarborane, to ensure a fair comparison with the bare CoSAN, was calculated using UV–Vis calibration curves, with HA‐Lys‐CoSAN **3** found to contain 1.31 ± 0.01 mmol CoSAN/g. Minor discrepancies may be due to inherent sample heterogeneity, although these measurements were performed in experimental and technical triplicates. The loaded materials were also tested using unloaded HA–lysine conjugates as a control. The results, presented in **Figure** **4**, confirm the successful and reproducible incorporation of COSAN into the HA‐based matrix, maintaining the cytotoxicity observed in our previous study for CoSAN alone under the same testing conditions.^[^ ^12^ ^]^ Selectivity was observed between the HDF and MDA‐MB‐231 cell lines, as previously reported, with HDF cells exhibiting greater resilience to the treatment than MDA‐MB‐231 cells. As anticipated, no significant differences were observed between CoSAN and HA‐Lys‐CoSAN **3** after 72 h of incubation, demonstrating the potential of noncovalent electrostatic loading. Cell count and % cell death after 72 h of treatment were investigated using the Operetta^TM^ high‐throughput microscopy system and Hoechst/propidium iodide staining. Using cell viability data, IC_50_ values for CoSAN and Ha‐Lys‐CoSAN 3 were measured to be 23 and 17 μM, respectively, for MDA‐MB‐231 cells after 72 h incubation (see Figure S9 and S10, Supporting Information). Compared to HDFs, the values up to 400 μM concentration are far from reaching 50% inhibition, showing clearly the higher activity toward TNBC cells. IC_50_ for CoSAN is aligned with previously reported data using MDA‐MB‐231 cells, with an IC_50_ after 24 h incubation of 137 μM.^[^ ^39^ ^]^

Additional details of the toxic effects of CoSAN on MDA‐MB‐231 cells were apparent. Likewise, HA–lysine **2** alone exhibited no measurable toxicity, highlighting its biocompatibility and suitability as a delivery platform. Importantly, the incorporation of CoSAN into HA‐Lys had no obvious cytotoxic effect compared to the inherent cytotoxicity of CoSAN itself, indicating that the noncovalent loading strategy preserved the native bioactivity of the parent compound. This was a desired consequence of the design of the delivery system, which aimed to change the properties of the parent compound as little as possible while also providing a means of administering the compounds with a biocompatible drug delivery system. Cell death and cell count measurements provided further insights into the behavior of CoSAN and HA‐Lys‐CoSAN **3**. Of relevance, there are marked differences in cell counts between CoSAN and HA‐Lys‐CoSAN **3**, indicating that at concentrations above 25 μM, the HA backbone provides a synergistic effect with CoSAN that causes a decrease in the number of cells. The differences between % cell viability data and % cell death can also be explained due to the known cytostatic effects that metallacarboranes can exert on different cell lines, as was shown in previous literature for HEK293, HeLa, and 3T3 cells.^[^ ^40^ ^]^ After analyzing the cytotoxicity profile of CoSAN, which remained unchanged upon loading to HA‐Lys, the next objective was to investigate the cellular internalization and ensure that it remained largely unchanged.

**Figure 5:**
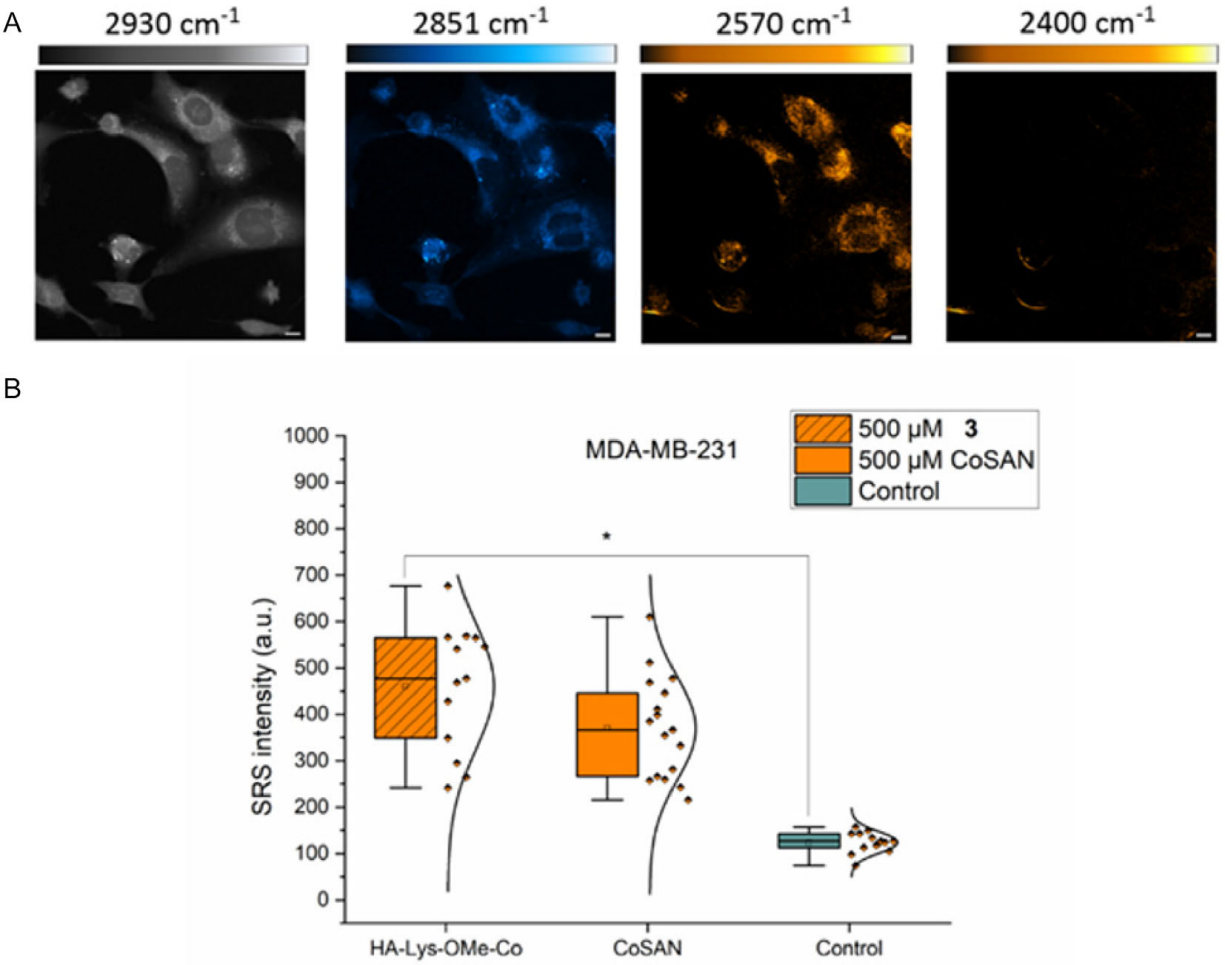
A) SRS images of MDA‐MB‐231 cell lines following treatment with 500 µM **3** at 2930 cm^*−*1^ (CH_3_, proteins), 2851 cm^*−*1^ (CH_2_, lipids), 2570 cm^*−*1^ (B–H), and 2400 cm^*−*1^ (off‐resonance). Look‐up tables: 2930 cm^*−*1^ (grey scale, 0–3000 a.u.), 2851 cm^*−*1^ (cyan hot, 0–3000 a.u.), 2570 cm^*−*1^ (orange hot, 0–2500 a.u.), 2400 cm^*−*1^ (orange hot, 0–2500 a.u.). Scale bars: 10 μm. B) Measured SRS intensities in MDA‐MB‐231 cell line. Data were extracted using ImageJ software, using a 2851 cm^*−*1^ image as a mask, and the measurements were set to the images at 2570 cm^*−*1^, which had been background subtracted using the images acquired at 2400 cm^*−*1^. Selections were made around the nucleus and cytoplasm regions of the cell using the ImageJ selection tool to measure the intracellular distribution. *p ≤ 0.05 (Student's t‐test).

Raman microscopy has recently garnered attention as a useful tool for imaging carboranes and metallacarboranes in vivo.^[^ ^41^ ^,^ ^42^ ^]^ Stimulated Raman scattering (SRS) stands out above other Raman techniques, as it combines high spatial resolution and fast image acquisition, which presents a higher compatibility with complex cellular imaging.^[^ ^43^ ^]^ In addition, SRS has successfully been used in metallacarboranes to image drug uptake in HeLa cells.^[^ ^44^ ^]^ To assess intracellular uptake, HA‐Lys‐CoSAN **3** at a concentration of 500 µM was investigated on MDA‐MB‐231 cell lines for 15 min, followed by SRS imaging. The resulting images are shown in **Figure** **5**.

The analysis of SRS intensity after 15 min of incubation with HA‐Lys‐CoSAN showed a similar value to that of CoSAN, and significantly to the control at >95% confidence. We quantified the mean SRS signal per cell at 2570 cm^−1^ as a means to compare the uptake. This indicated that, with respect to the control cell population, both HA‐Lys‐CoSAN **3** and CoSAN led to a notable increase in SRS signal intensity at 2570 cm^−1^. However, a similar mean value was obtained for both treatment conditions, which suggested that the uptake was equivalent. There was no significance found between SRS intensities within the cells following treatment with CoSAN and HA‐Lys‐CoSAN **3.** However, a general trend indicates the higher internalization of **3** as HA conjugation facilitates improved cellular uptake of metallacarboranes, and this can be traced back to the higher % cell death of HA‐Lys‐CoSAN **3** compared to the bare CoSAN.

### 2.3 Release Study of HA‐Lys‐CoSAN 3

**Figure 6:**
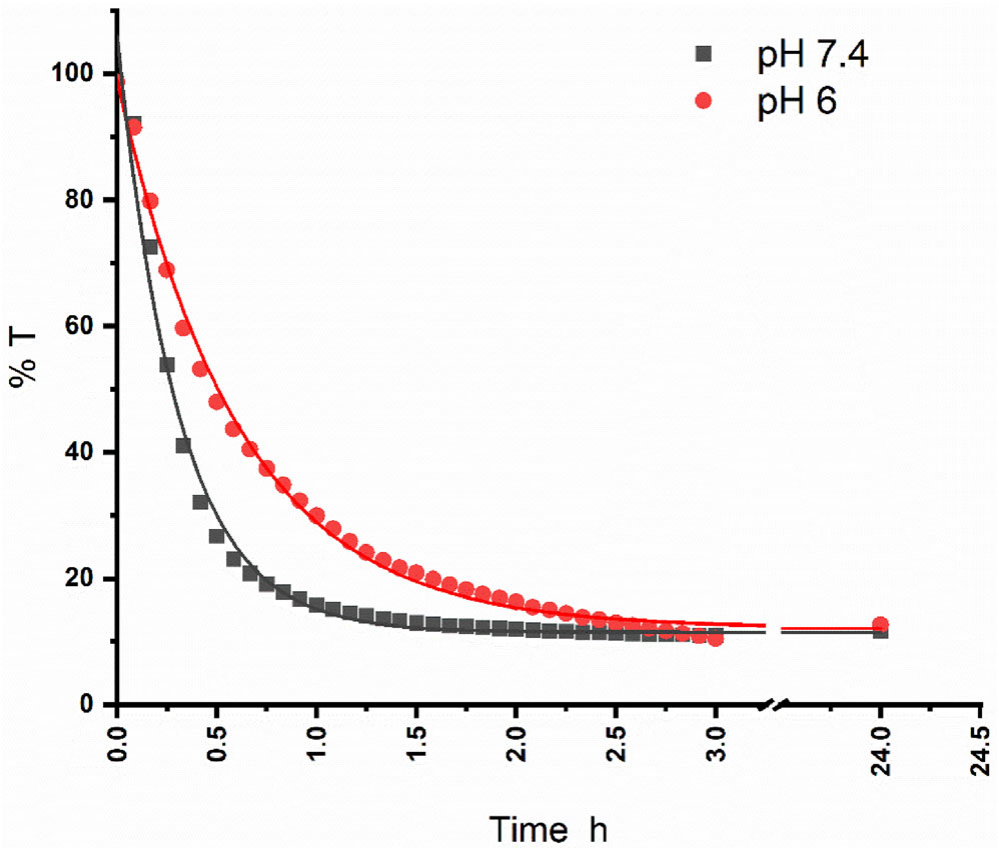
Measured %*T* every 30 min between 0 and 3 h, and at 24 h of HA‐Lys‐CoSAN **3** at 37 °C, at pH 7.4, and 6.0, with the UV–Vis probe measuring outside the dialysis membrane.

The release of CoSAN from **3** was studied using UV–Vis spectroscopy combined with the dialysis method (Figure S11 and S12, Supporting Information). The conjugated material HA‐Lys‐CoSAN **3** was placed inside a dialysis membrane with a 14 kDa molecular weight cut‐off and suspended in PBS to mimic physiological conditions. This membrane retained the large conjugates inside while allowing only the released CoSAN to diffuse out. A UV–Vis spectrophotometer was placed in the external PBS solution to detect the release of the drug by measuring the changes in absorbance over time. The spectra at *t* = 0, 3, and 48 h are provided in Figure S9, Supporting Information, to show the increase in intensity of the expected peak at 280 nm for free CoSAN (the inset in Figure S9, Supporting Information, also shows the UV–Vis spectrum of an aqueous solution of CoSAN as a reference). The release kinetics were measured over 24 h at 37 °C using either pH 7.4 or 6.0 (to mimic the tumor microenvironment) and comparing the kinetics in the two different pH solutions (**Figure** **6**). Fitting the curves to a monoexponential decay led to kinetic constants of 18.4 and 36.5 h^−1^, respectively. The slower release kinetics at pH 6.0 can be understood by a higher proportion of protonated Lys (H^+^Lys), leading to a stronger electrostatic interaction with CoSAN in a more acidic medium. However, when the pH increased to 7.4, the proportion of deprotonated Lys increased (pKa of Lys is 10.5), leading to weaker electrostatic interactions with CoSAN, thus releasing the anion faster into the solution. This also suggests that in cancer tumor microenvironments, where the pH is usually lower, the release occurs more gradually.^[^ ^45^ ^]^

## 3 Conclusion

In this study, we successfully developed a noncovalent HA‐Lys‐CoSAN composite that preserves the intrinsic bioactivity of CoSAN while leveraging the HA biopolymer backbone for selective targeting of TNBC cells via CD44 receptor‐mediated uptake. The composite was synthesized and thoroughly characterized using multiple analytical techniques. Elemental mapping confirmed the effective incorporation of CoSAN into HA‐Lys matrices, and Raman imaging indicated a modest enhancement in cellular uptake, which corresponded with increased cytotoxic activity. The structural integrity of the CoSAN cluster was maintained, as no cage functionalization was required. The link between CoSAN and HA was facilitated by electrostatic interactions between the positively charged lysine and negatively charged CoSAN, enabling efficient drug loading and resulting in greater growth inhibition compared to free CoSAN. Controlled release studies demonstrated a sustained release profile under acidic conditions, supporting the potential for tumor‐selective drug delivery. Moreover, cellular assays confirmed HA as an effective carrier for metallacarboranes, improving both uptake and therapeutic selectivity. Overall, these results underscore the potential of HA‐based delivery platforms for metallacarborane compounds and justify further investigation into their application in targeted cancer therapy.

## Conflict of Interest

The authors declare no conflict of interest.

## Author Contributions

**Neville Murphy**: data curation (lead); formal analysis (equal); investigation (lead); methodology (equal); writing—original draft (lead). **Roberto González‐Gómez**: conceptualization (supporting); data curation (equal); methodology (equal); supervision (supporting); visualization (equal); writing—review & editing (equal). **Nivethitha Ashok**: investigation (supporting); visualization (supporting); writing—review & editing (equal). **Enda O’Connell**: data curation (supporting); formal analysis (supporting); investigation (supporting). **Howard Fearnhead**: data curation (supporting); formal analysis (supporting); investigation (supporting). **William J. Tipping**: data curation (supporting); formal analysis (supporting); investigation (supporting); supervision (supporting); visualization (supporting); writing—review & editing (supporting). **Karen Faulds**: data curation (supporting); formal analysis (supporting). **Wenming Tong**: data curation (supporting); investigation (supporting). **Abhay Pandit**: formal analysis (supporting); investigation (supporting); supervision (supporting); writing—review & editing (supporting). **Róisín M. Dwyer**: investigation (supporting); resources (supporting); supervision (supporting); writing—review & editing (supporting). **Duncan Graham**: funding acquisition (supporting); resources (supporting); supervision (supporting); writing—review & editing (supporting). **Pau Farràs**: conceptualization (lead); funding acquisition (lead); project administration (lead); supervision (lead); writing—original draft (supporting); writing—review & editing (equal).

## Supporting information

Supplementary Material

## Data Availability

The data that support the findings of this study are available in the *supplementary material* of this article.
