# Supplementary material for: Functionalizing Injectable Hydrogels with Cobalt‐Based Metallacarboranes for Targeted Delivery in Triple‐Negative Breast Cancer
Source: Chembiochem. 2025 Oct 7;26(21):e202500589. doi: 10.1002/cbic.202500589 (PMC12596922; doi:10.1002/cbic.202500589)
Supplement: Supplementary file 1 — Supplementary Material [file CBIC-26-e202500589-s001.pdf]

SUPPORTING INFORMATION

Functionalizing injectable hydrogels with cobalt-based metallacarboranes for targeted delivery in triple-negative breast cancer

Neville Murphy[a,b], Roberto González-Gómez[a], Nivethitha Ashok[a,b], Enda O'Connell[c], Howard Fearnhead[d], William J. Tipping[e], Karen Faulds[e], Wenming Tong[a], Abhay Pandit[b], Róisín M. Dwyer[b,f], Duncan Graham[e], Pau Farràs*[a,b]

[a] N Murphy, R González-Gómez, N Ashok, W Tong, P Farràs
School of Biological and Chemical Sciences, Ryan Institute, University of Galway, H91 CF50 Galway, Ireland.
E-mail: pau.farras@universityofgalway.ie

[b] N Murphy, N Ashok, A Pandit, P Farràs
CÚRAM Research Ireland Centre for Medical Devices, University of Galway, H91 W2TY Galway, Ireland

[c] E O'Connell
Genomics and Screening Core, University of Galway, H91 W2TY Galway, Ireland

[d] H Fearnhead
Pharmacology and Therapeutics, University of Galway, Galway H91 TK33, Ireland

[e] W J Tipping, K Faulds, D Graham
Centre for Molecular Nanometrology, Department of Pure and Applied Chemistry, Technology and Innovation Centre, University of Strathclyde, Glasgow G1 1RD, United Kingdom

[f] RM Dwyer
School of Medicine, Lambe Institute for Translational Research, University of Galway, H91 TK33 Galway, Ireland

Table of contents

Materials and methods 2
- **Synthesis of compounds and polymers** 2
- **Methods** 2
- **Cell Culture** 3
- **Cell Assay procedure** 4

Schemes 5
Figures 6
References 12

Materials and methods

Synthesis of compounds and polymers

o-$C_2B_{10}H_{12}$ was purchased from Katchem and used as received. Hyaluronic acid was obtained from LifeCore Biomedical. All other solvents and organic and inorganic salts were purchased from Across Organics, TCI, or Sigma-Aldrich at analytical reagent grade and used as received.

*HA-L-lys(Boc)-OMe (**1**)*

40.0 mg HA 60kDa (92.7 µmol) was added to a 4 mL, 10 mM PBS solution and gently stirred after the polymer was fully dissolved (~ 40 min) to prevent shear. A 1 mL H_2O solution containing 25.6 mg DMTMM (92.7 µmol) and 55.0 mg L-lys (Boc)-OMe.HCl (185.4 µmol) was added. The pH was adjusted to 6 using 1 M HCl. This was stirred overnight at 30 °C, and the polymer was precipitated with EtOH. The white film was steeped in methanol for ten minutes, then carefully washed and decanted with MeOH, EtOH, and acetone three times each. It was then dialysed in deionised water for 24 h to remove the salts. The solvent was removed *in vacuo*. The mixture was dried *in vacuo* to obtain 49.3 mg **1**. ^1H NMR (D_2O, 500 MHz): δ = 4.4 (2H, dd), 3.82 (3H, s), 3.8-3.2 (8H, m), 2.94 (2H, t), 1.88 (3H, s), 1.31 (9H, s). FT-IR; 3370 cm^{-1} (s, b) 2926 cm^{-1} (w, b) (C−H) 2866 cm^{-1} (m) 2762 cm^{-1} (m) 2478 cm^{-1} (w) 1686 cm^{-1} (m, C=O) 1578 cm^{-1} (w) 1578 cm^{-1} (w) 1530 cm^{-1} (m) 1457 cm^{-1} (w) 1402 cm^{-1} (m) 1367 cm^{-1} (m) 1320 cm^{-1} (w) 1274 cm^{-1} (w) 1249 cm^{-1} (m) 1165 cm^{-1} (w).

*HA-L-lys-OMe (**2**)*

To 40.0 mg of **1** was added to 1.0 mL 1 M HCL, and 0.5 mL TFA was added, and the mixture was allowed to dissolve. The mixture was stirred at 30 °C for 5 h, and then neutralised with 1 M NaOH in an ice bath. The solvent was removed *in vacuo,* and the transparent film was steeped in MeOH for 10 min, then carefully washed and decanted with MeOH, EtOH, and acetone three times each. It is then dialysed vs. deionised water for 24 h to remove salts. The film was dried under vacuum to obtain 56.4 mg of HA-L-lys-OMe **2**. ^1H NMR (D_2O, 500 MHz): δ = 4.4 (2H, dd), 3.82 (3H, s), 3.8-3.2 (8H, m), 2.88 (2H, t), 1.89 (3H, s). FT-IR; 3348 cm^{-1} (s, b) 2954 cm^{-1} (w, b) (C−H) 1674 cm^{-1} (s) (C=O) 1432 cm^{-1} (m) 1374 cm^{-1} (m) (O−H) 1318 cm^{-1} (m) 1199 cm^{-1} (s) 1133 cm^{-1} (s) 1038 cm^{-1} (s).

*HA-L-lys-OMe[CoSAN] (**3**)*

Compound 2 (40.0 mg, 8.3 µmol) was added to 1.0 mL of 1 M HCL and allowed to dissolve. A concentrated solution of Na[CoSAN] (1 mM) was added dropwise to the mixture. The resulting orange precipitate was filtered and washed with 1M HCl (3 × 2 mL). The mixture was dried *in vacuo* to obtain 62.8 mg **3**. ^1H NMR (D_2O, 500 MHz): δ = 3.7 (3H, s), 3.9–3.2 (HA, m), 2.11 (2H, t), 1.83 (2H, t), 1.9 (3H, s), ^{11}B NMR (D_2O, 160.4 MHz): δ = 4.16 (s, 2B, 1J), 0.56 (s, 2B, 1J), -7.4 (d, 8B), -18.11 (s, 4B, 1J), -23.85 (s, 2B, 1J). FT-IR; 3367 cm^{-1} (s, b) 3255 cm^{-1} (s, b) (C−H) 2530 cm^{-1} (m) (B−H) 1726 cm^{-1} (m) (C=O) 1641 cm^{-1} (m) (C=O) 1564 cm^{-1} (m) 1432 cm^{-1} (m).

Methods

^1H, ^{11}B, and ^{13}C NMR spectroscopy was performed on a Varian 500 MHZ 54 mm AR spectrometer. The spectra for ^1H, ^{13}C, and ^{11}B were recorded at 500, 125, and 160.4 MHz, respectively. Trimethylsilane (TMS) was used as a reference for ^1H and ^{13}C, and boron trifluoride diethyl etherate was used for ^{11}B. The deuterated solvents used included D_2O and C_2D_3N. The solvents used are stated in the spectra captions.

UV-Vis absorbance measurements were performed using a Varian Cary 50 UV scan spectro-photometer. Transmission measurements were performed using a Cary 5000 scan spectro-photometer equipped with a quartz probe with a 1 cm path length (Agilent). The release of metallacarboranes from HA formulations was measured using a variation of the dialysis method, which placed the materials within a dialysis membrane (Merck) in solution with a 14 kDa cut-off, preventing larger polymer molecules from escaping and only detecting released fragments. Detection

was performed using a UV-Vis probe outside the dialysis bag but within the dialysis chamber to measure the change in transmittance over time at regular intervals.

Nonlinear curve fitting of the release profiles at pH 6.0 and 7.4 was performed using a single exponential decay model (ExpDec1: $y = A1*\exp(-x/t_1) + y_0$).

For pH 6.0, the fitting parameters were as follows:
$y_0 = 12.1537 \pm 0.4427$
$A1 = 86.93186 \pm 0.96027$
$t1 = 36.48277 \pm 0.88783$
The model yielded an R^2 of 0.9962 (adjusted $R^2 = 0.99598$).

For pH 7.4, the fitting parameters were
$y_0 = 11.52572 \pm 0.51527$
$A_1 = 94.43456 \pm 2.03153$
$t_1 = 18.41066 \pm 0.72825$
The fit showed an R^2 of 0.98757 (adjusted $R^2 = 0.98686$).

Raman spectra were acquired on a Renishaw InVia Raman microscope equipped with a 532 nm Nd:YAG laser providing a maximum power of 45 mW using a 1800 l/mm grating, a 633 nm HeNe laser providing a maximum power of 17 mW using a 1200 l/mm grating, and a 785 nm diode laser providing a maximum power of 300 mW using a 1200 l/mm grating.

Raman spectra of fixed cells were acquired using a Witec Alpha 500 confocal laser Raman microscope in upright configuration with a 100-micron fibre Toptica 785 nm laser, a 600 grooves/mm diffraction grating and an Andor Idus CCD camera (Andor Technology Ltd., Belfast, Ireland). The spatial resolution of the laser was ~ 1 µm. The system was calibrated to a standard silicon peak at 520 cm^{-1}. The spectral resolution for all the measurements was 1.5 cm^{-1}. The spectra were treated for cosmic ray removal and processed using background subtraction and Savitzky–Golay smoothing. (WITEC Project 4, software version 4.1).

IR spectra (4000–650 cm^{-1}) were recorded using a PerkinElmer 16PC FT-IR spectrometer with KBr reference.

SEM (15 keV) and EDX analyses were performed at the “Centre for Microscopy and Imaging” at the University of Galway, Ireland. SEM-EDX measurements were carried out on a Hitachi S-4700 SEM with an EDX spectrometer, and EDX spectra were recorded using Oxford Instruments INCA Energy EDX detector and Bruker XFlash 6160 EDX detector (20 keV). Samples were drop-cast onto clean glass slides or silicon wafers (5 mm × 5 mm) in cyclohexane, allowed to dry, and then coated with gold in a Quorum Q150R ES plus sputter coater before SEM measurements.

Cell Culture

To ensure the availability of nutrients for MDA-MB-231 and HDF cells, and the removal of waste for optimal growth conditions, the cell medium (DMEM-Gibco, Thermofisher) was replenished three times a week. Prior to feeding, supplemented media were warmed to 37 °C in a water bath to prevent temperature shock to the cells. Cells were observed in bright field under a light microscope to evaluate cell density and morphology, as well as checking for indicators of contamination (e.g., turbidity, particulate matter, or change in pH). The cell culture flask, fitted with a filter lid to accommodate gas exchange, was then returned to the incubator at 37 °C and 5% CO_2. To avoid senescence and maintain the log phase of growth within the cell culture at 80-90% confluency, the cells were sub-cultured.

Cell Assay procedure

Cells were seeded at densities of 5,000 and 10,000 cells per well, respectively, in 96-well plates. These were then left to attach overnight at 37 °C in the respective culture media. This media was then replaced with relevant drug dilutions made up in complete culture media and incubated for stated treatment periods. This was then replaced by fresh media to which MTS reagent (Abcam) [46] was added and incubated for 3 hours, followed by reading absorbance at 490 nm wavelength (Viktor X5 plate reader (PerkinElmer)). Operetta CLS high content system (PerkinElmer) was used for live/dead and cell count assay, with Hoechst and propidium iodide stains (Merck).

Cellular SRS imaging procedure

The procedure for analysing cellular uptake of metallacarboranes through SRS imaging was as follows. Cells were seeded at 150,000 cells per mL on high precision glass coverslips (#1.5H thickness, 22 × 22 mm, Thorlabs) in a 6-well plate in Dulbecco's modified eagle medium (DMEM) at a concentration of 1.5×10^5 cells per mL and incubated at 37 °C and 5% CO_2 for 24 h prior to treatment. The next day, the coverslips were carefully removed from the plates with tweezers, and placed cell-face down onto a microscopy slide with 7.5 µL of a 500 µM solution of the relevant metallacarborane species in the relevant media, and incubated at 37 °C, 5% CO_2 for 15 min; this time frame was chosen as longer incubations times caused significant levels of cell death, and it was found that the uptake was fast.

For SRS imaging, an integrated laser system (picoEmerald™ S, Applied Physics & Electronics, Inc.) was used to produce two synchronised laser beams at 80 MHz repetition rate. A fundamental Stokes beam (1031.4 nm, 2 ps pulse width) was intensity modulated by an electro-optic-modulator (EoM) with >90% modulation depth, and a tuneable pump beam (700–960 nm, 2 ps pulse width, <1 nm (10 cm^{-1}) spectral bandwidth) was produced by a built-in optical parametric oscillator. The pump and Stokes beams were spatially and temporally overlapped using two dichroic mirrors and a delay stage inside the laser system and coupled into an inverted laser-scanning microscope (Leica TCS SP8, Leica Microsystems) with optimised near-IR throughput. SRS images were acquired using 40× objective (HC PL IRAPO 40×, N.A. 1.10 water immersion lens) with a 9.75–48 µs pixel dwell time over a 512 × 512 or a 1024 × 1024 frame. The Stokes beam was modulated with a 20 MHz EoM. Forward scattered light was collected by an S1 N. A. 1.4 condenser lens (Leica Microsystems). Images were acquired at 12-bit image depth. The laser powers measured after the objective lens were in the range 10–30 mW for the pump beam only, 10–50 mW for the Stokes beam only and 20–70 mW (pump and Stokes beams). The pump and Stokes beams were adjusted to image at 2930 cm^{-1} (CH_3, proteins, symmetric stretch) and 2851 cm^{-1} (CH_2, lipids, symmetric stretch) to determine the cells as regions of interest. The SRS intensity was then measured at 2570 cm^{-1} (B−H, symmetric stretch) to determine the relative uptake of the compounds, and finally at 2400 cm^{-1} to measure the background off-resonance signal. The spatial resolution of the system is ∼450 nm (pump wavelength = 792 nm).

Schemes

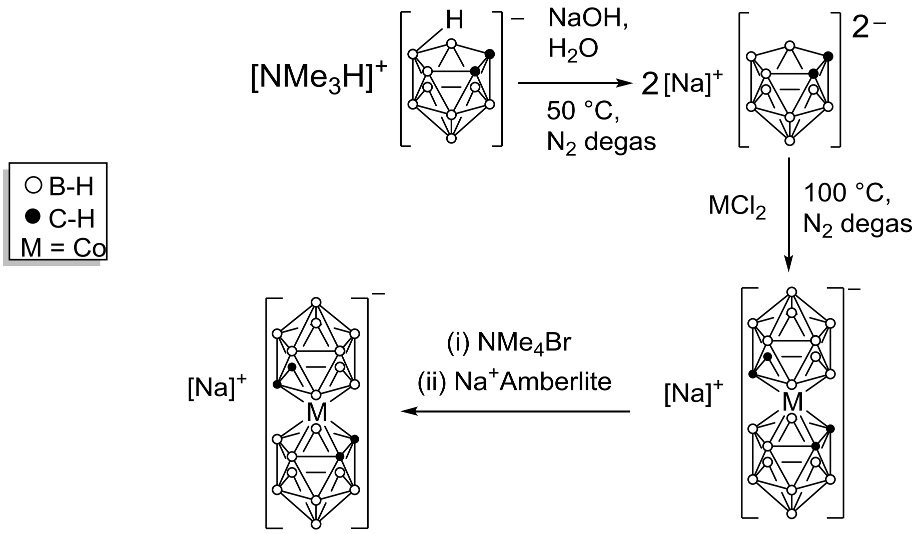

Scheme S1: Synthesis of CoSAN.

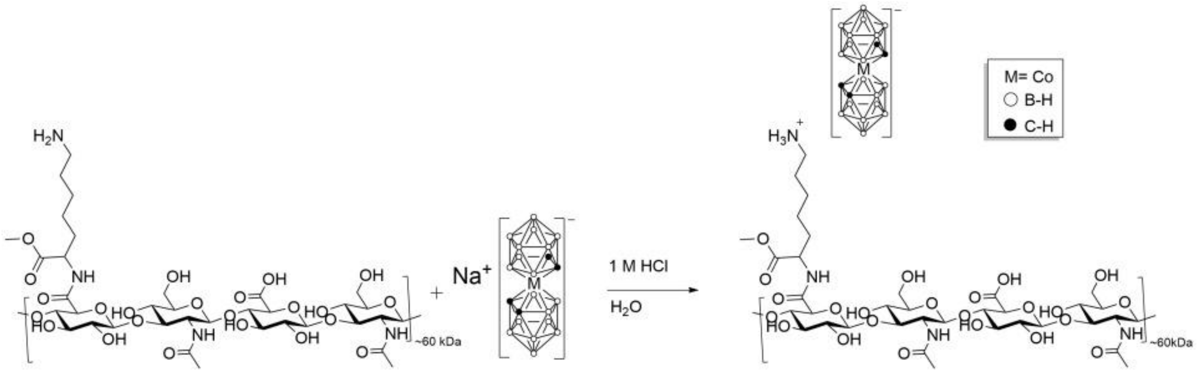

Scheme S2: Formation of non-covalently loaded metallacarborane-hyaluronic acid materials.

Figures

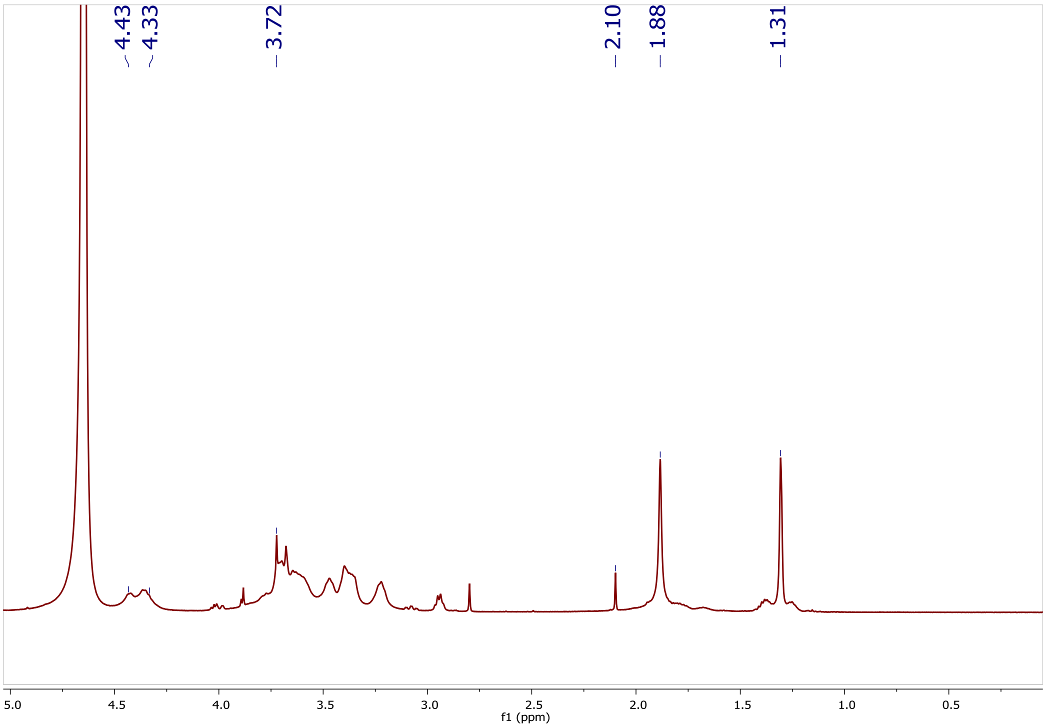

Figure S1: ^{1}H NMR spectrum of **1** in D_2O.

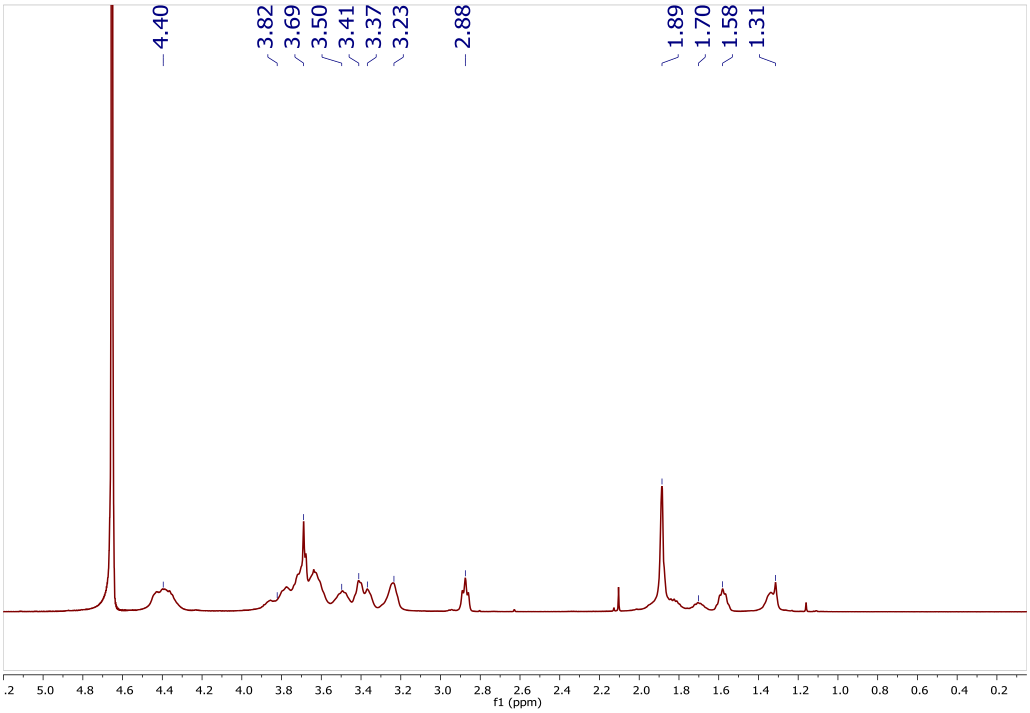

Figure S2: ^{1}H NMR spectrum of **2** in D_2O.

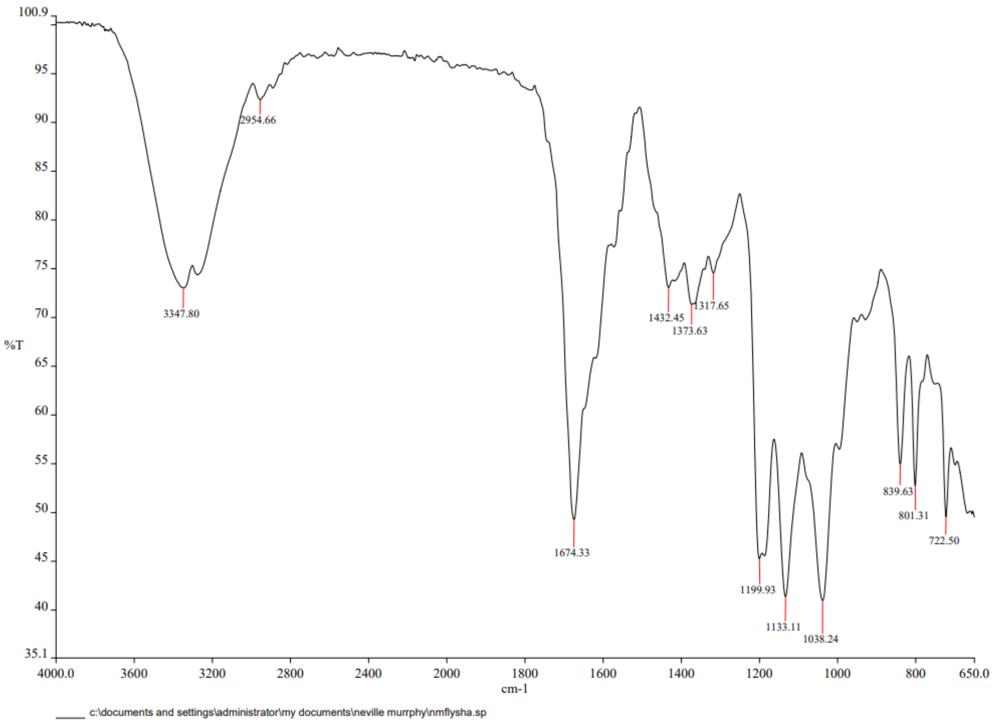

Figure S3: FTIR spectrum of **2** in D_2O.

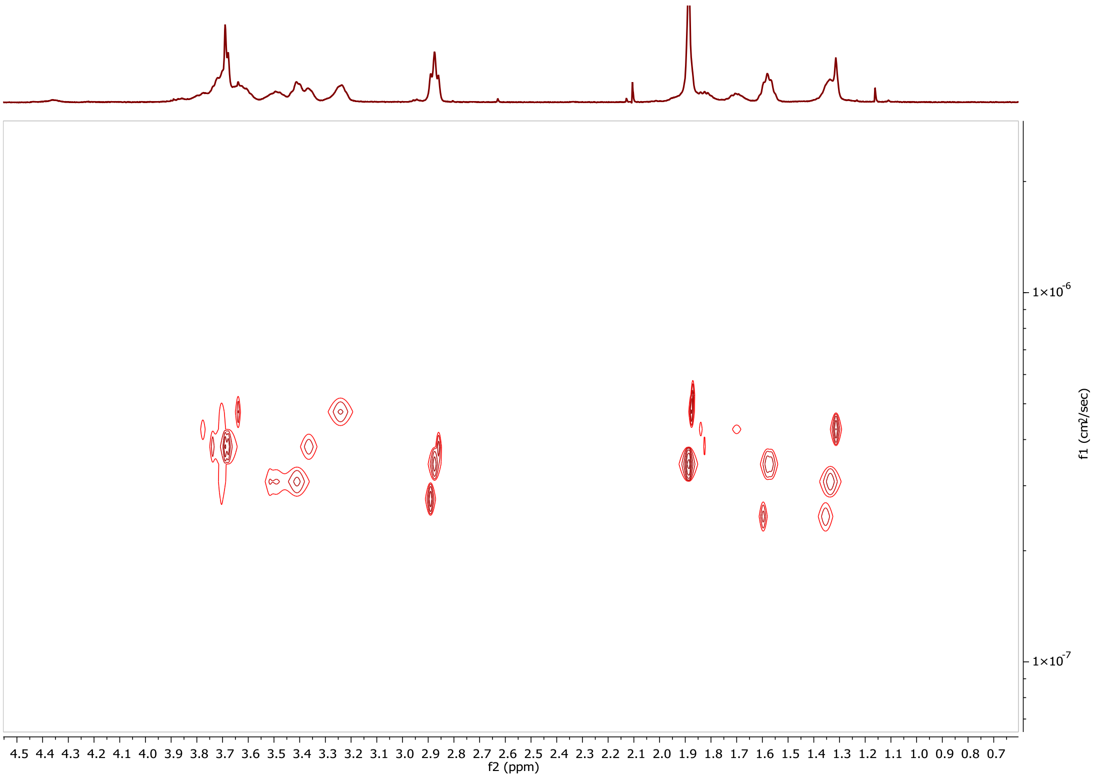

Figure S4: DOSY spectrum of **2** in D_2O.

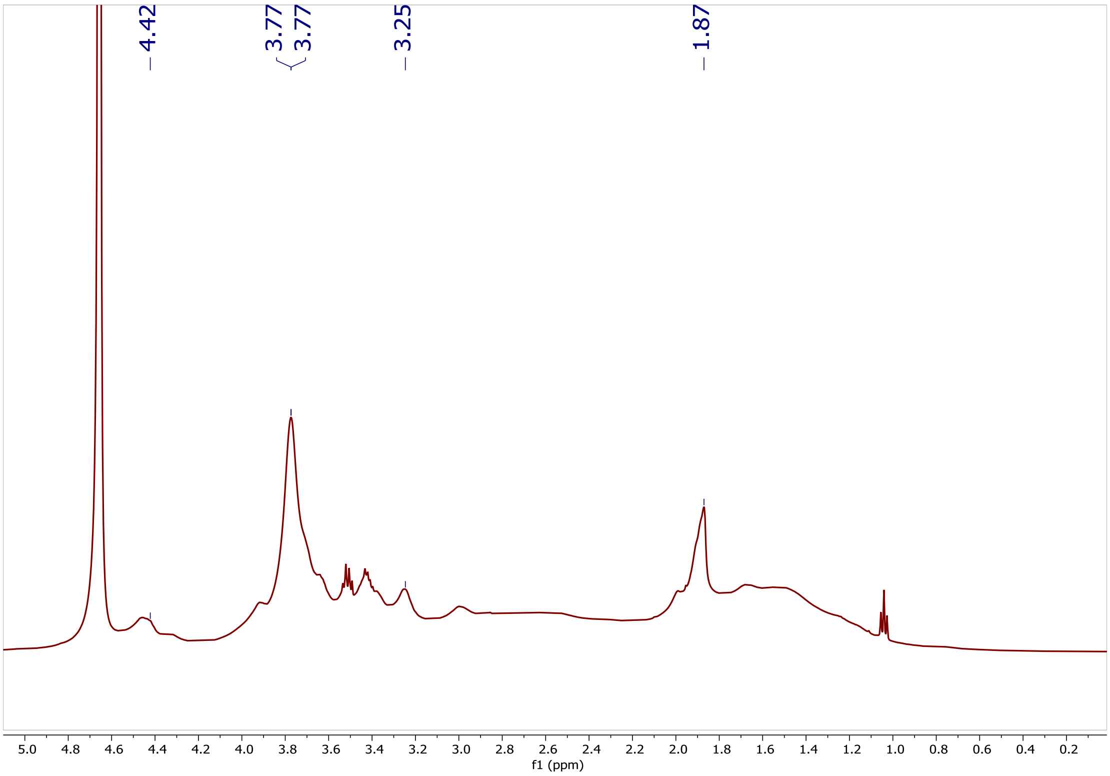

Figure S5: ^{1}H NMR spectrum of HA-Lys-CoSAN **3** in D_2O.

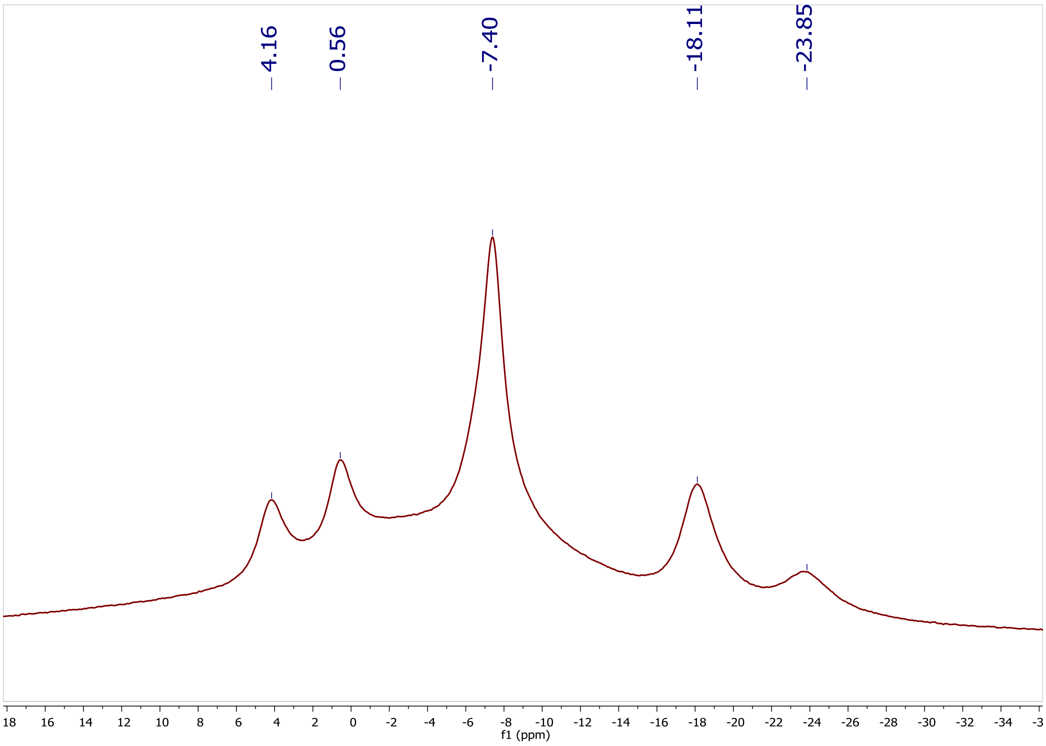

Figure S6: ^{11}B NMR spectrum of HA-Lys-CoSAN **3** in D_2O.

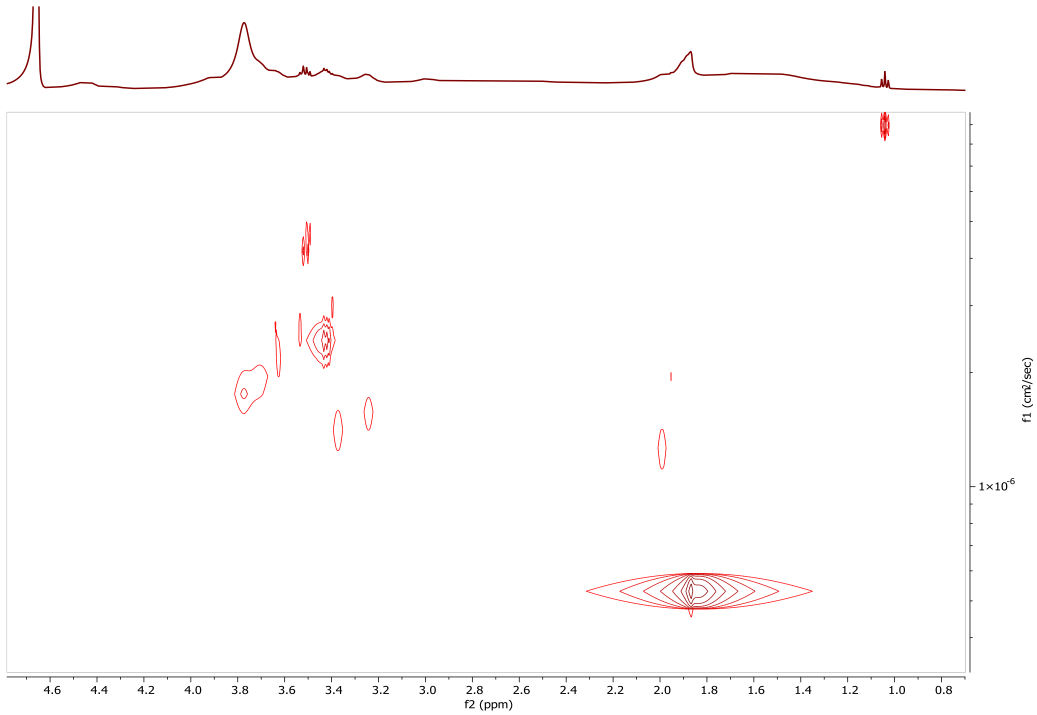

Figure S7: DOSY spectrum of HA-Lys-CoSAN **3** in D_2O.

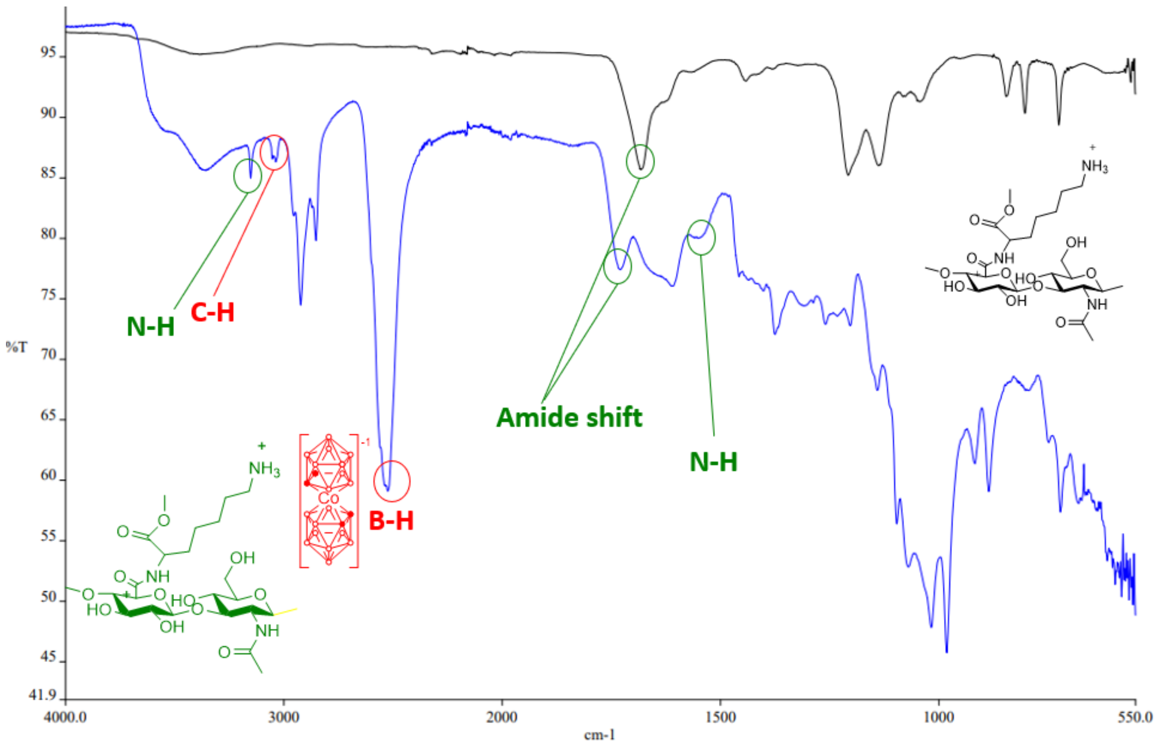

Figure S8: FTIR spectrum of HA (black) vs. HA-Lys-CoSAN **3** (blue).

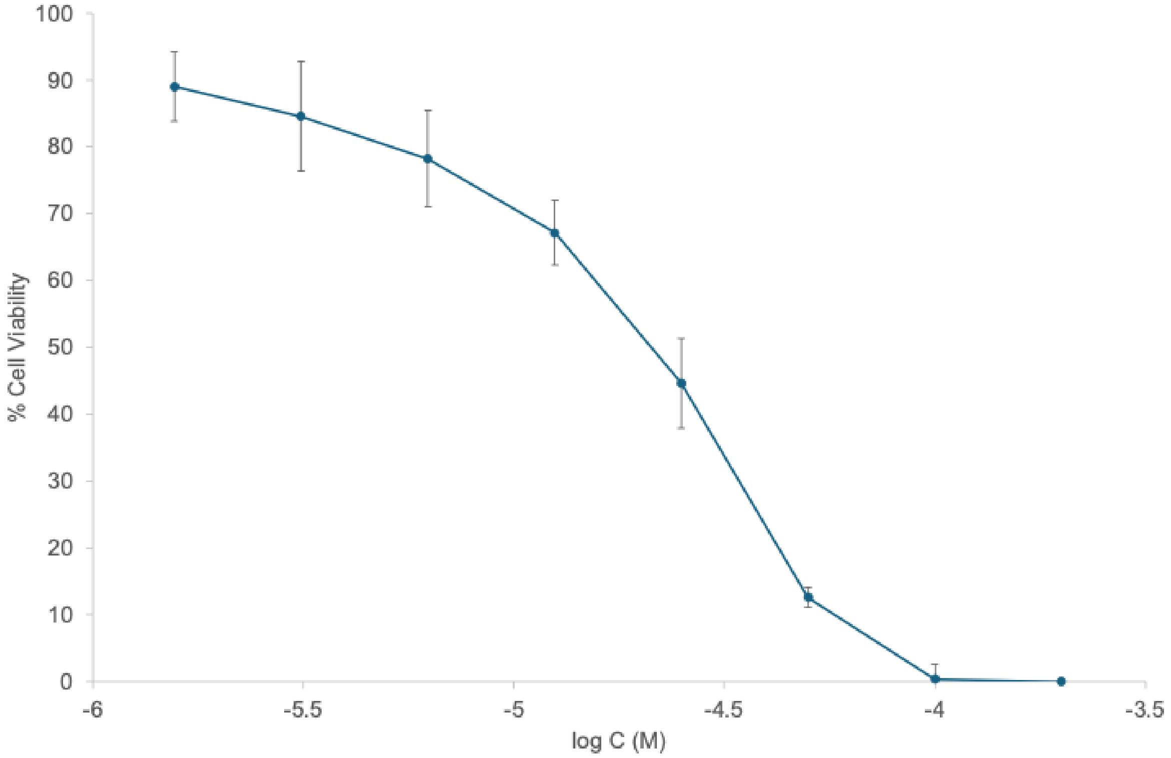

Figure S9: Dose-response curves obtained to calculate the IC_{50} values of Na[CoSAN] at 72 h incubation. Results are mean ± SD of three independent experiments.

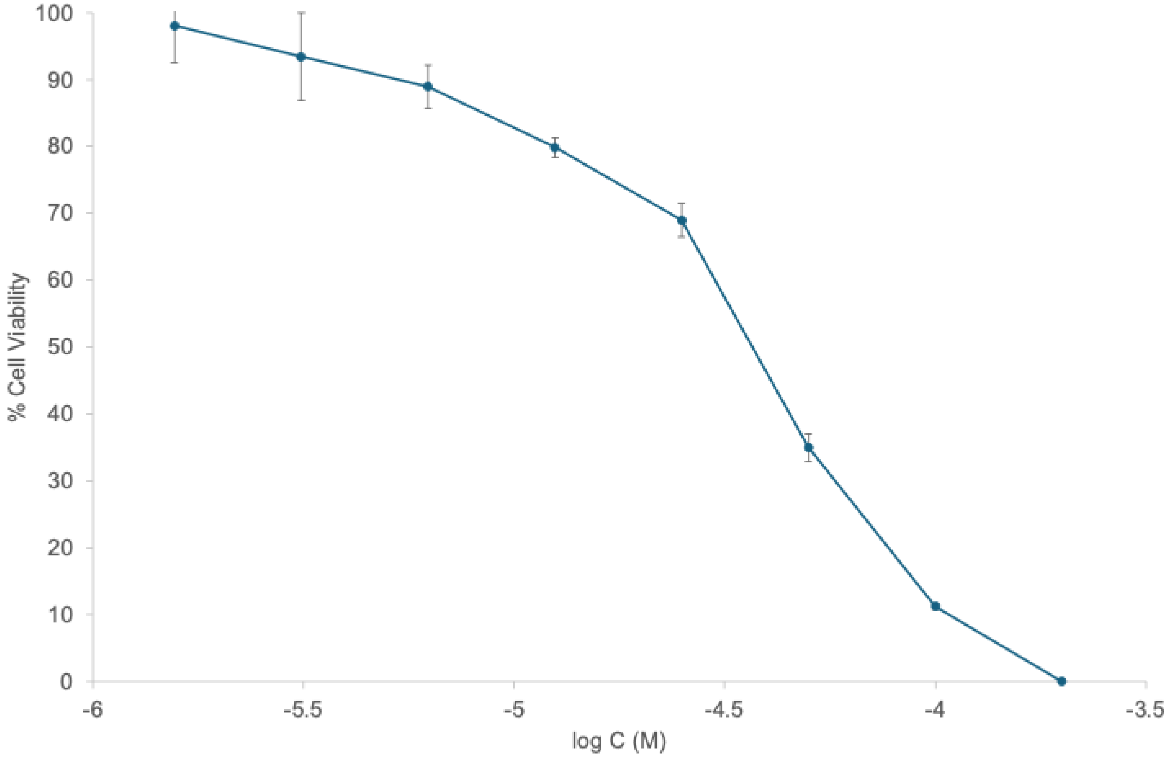

Figure S10: Dose-response curves obtained to calculate the IC_{50} values of HA-Lys-CoSAN **3** at 72 h incubation. Results are mean ± SD of three independent experiments.

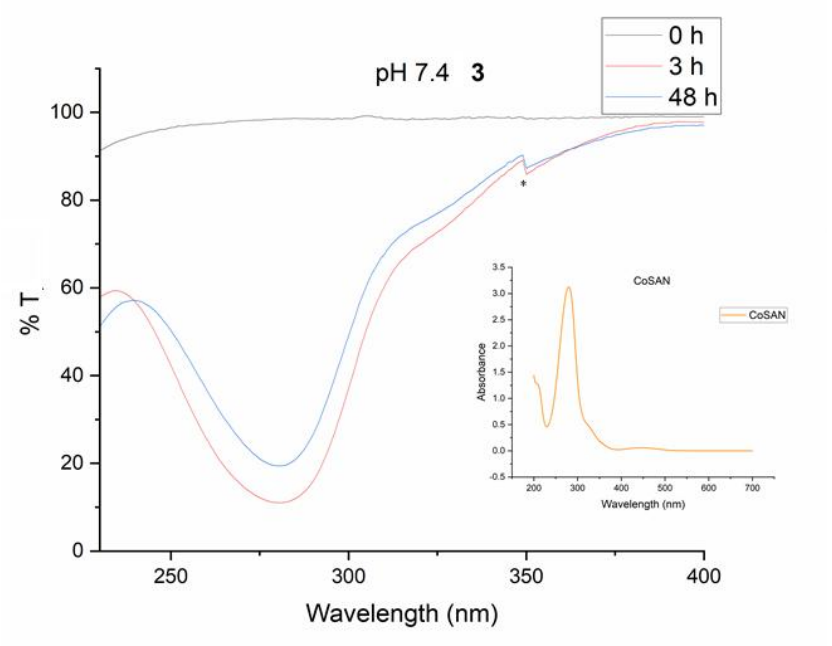

Figure S11: Measured %T at 0 h and 48 h of HA-Lys-CoSAN **3** at 37 °C, pH 7.4, with the probe measuring outside the dialysis membrane. * Denotes aberration from the grate changing in the UV-Vis. Absorbance spectrum of CoSAN overlayed for comparison.

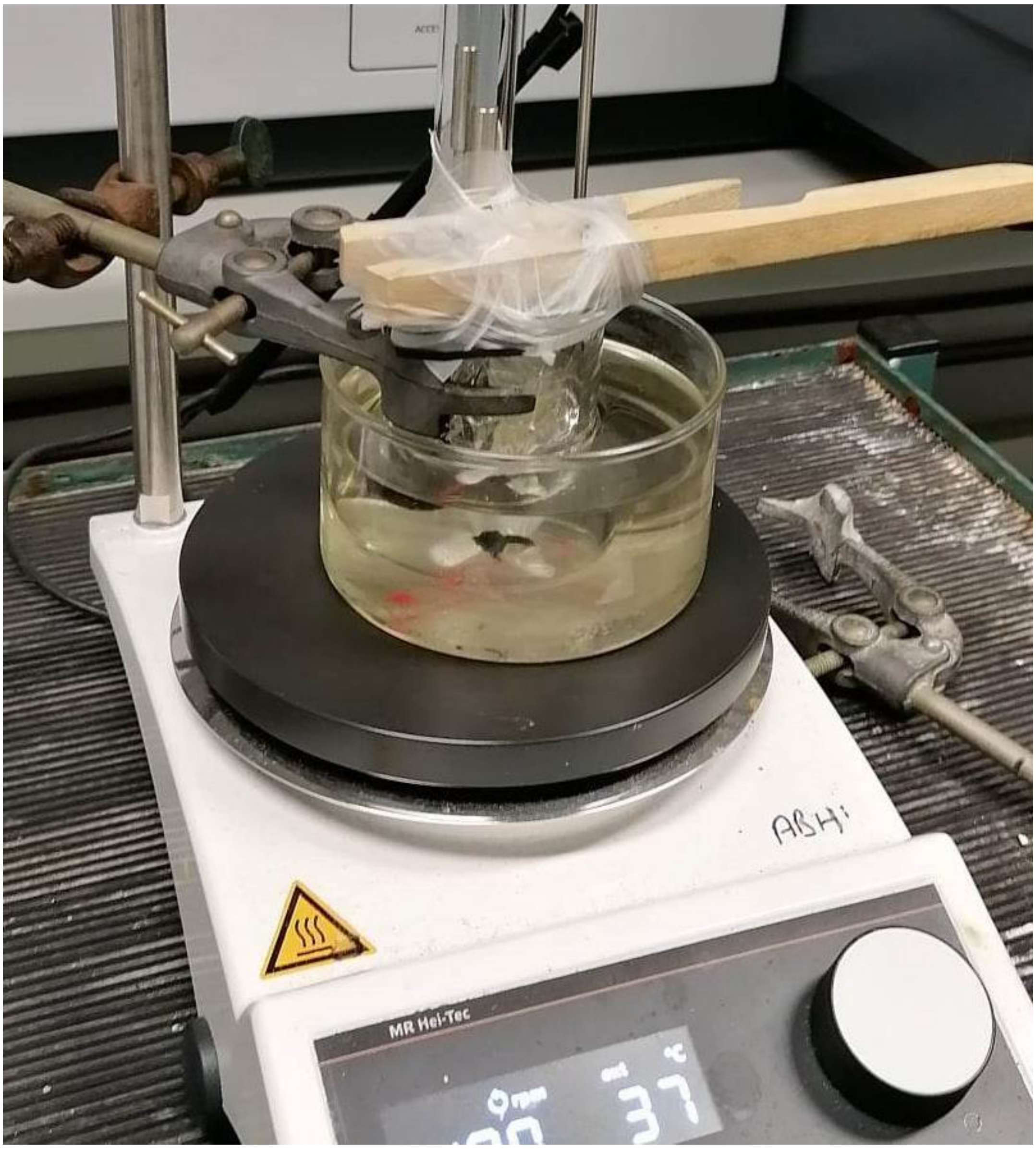

Figure S12: Image of dialysis method setup for release studies, using a UV-Vis fibre optic probe connected to a Cary 5000 UV-Vis-NIR.

References

[46] K. Berg, L. Zhai, M. Chen, A. Kharazmi, T. C. Owen, *Parasitol Res* **1994**, *80*, 235–239.
